# Prevalence of Hypertension and Associated Factors in Dire Dawa City, Eastern Ethiopia: A Community-Based Cross-Sectional Study

**DOI:** 10.1155/2019/9878437

**Published:** 2019-05-15

**Authors:** Hirbo Shore Roba, Addisu Shunu Beyene, Melkamu Merid Mengesha, Behailu Hawulte Ayele

**Affiliations:** ^1^Haramaya University, College of Health and Medical Science, School of Public Health, Harar, Ethiopia; ^2^Research Center for Generational Health and Ageing, School of Medicine and Public Health, Faculty of Health and Medicine, University of Newcastle, Australia

## Abstract

**Background:**

Hypertension is a major cardiovascular risk factor that is linked with fatal complications and is an overwhelming global challenge. Primary prevention is a key to control hypertension with identification of major risk factors. This study was aimed at assessing the prevalence and factors associated with hypertension.

**Methods:**

Community-based cross-sectional study was conducted among 903 adults aged 25 to 64 years in Dire Dawa City, East Ethiopia. Data were collected using World Health Organization (WHO) STEPwise approach to Surveillance (STEPS) for non-communicable disease (NCD) standard survey tool. Multivariate logistic regression models were used to identify relative effects of distal, proximal, and immediate risk factors of hypertension, and all statistical tests were declared significantly at* P*-value<0.05.

**Results:**

The average SBP and DBP were 124.98±17.18 mmHg and 78.92±10.13 mmHg, respectively. The prevalence of hypertension was 24.43% (95% CI: 21.57, 27.28). Majority (51.64%) of adults were not aware of their elevated blood pressure status. hypertension was significantly associated with the age group 30-44 (aOR 3.61, 95% CI: 2.0, 6.55), 45-54 (aOR 5.36, 95% CI: 2.62, 10.91), and 55-64 (aOR 9.38, 95% CI: 4.73, 18.59), being unemployed (aOR 1.68, 95%CI: 1.03, 2.77), ever smoking (aOR 1.89, 95% CI: 1.04, 2.23), having abdominal obesity (aOR 1.72, 95% CI: 1.13, 2.64), and BMI≥25 kg/m^2^ (aOR 1.48, 95%CI: 1.01, 2.15).

**Conclusion:**

Moderately high prevalence of hypertension was observed among adults in study setting demonstrating a major public health problem. Majority of adults with hypertension in study setting were not aware of their elevated BP status highlighting the burden of the hidden morbidity and subsequent complications. Community level intervention and routine assessment of sociodemographic, behavioral, and biophysiological risk factors, screening, and diagnosis of NCDs should be institutionalized to address the occult burden.

## 1. Background

Hypertension is a progressive cardiovascular disease which arises from complex and interrelated etiologies and is characterized by persistently elevated systemic blood pressure [1]. It is associated with fatal complications like coronary artery disease, cerebrovascular accidents, congestive heart failure, renal failure, and peripheral arterial diseases [1–3]. High blood pressure, systolic blood pressure (SBP) greater than or equal to 140 mmHg, or diastolic blood pressure (DBP) greater or equal to 90 mmhg is an overwhelming global challenge which is the third contributor of high disability adjusted life-years [4].

Globally, in 2015, 1.13 billion adults had hypertension [5] and it contributed about 211.8 million disability adjusted life-years (DALYS) [6]. The mean blood pressure is increasing in low- and middle-income countries while being declining or remaining unchanged in developed countries during period of 1975 to 2015. In 2015, sub-Saharan Africa had the highest burden of raised blood pressure compared with countries with the highest income [5].

The prevalence of hypertension in sub-Saharan Africa estimated in 2008 was 13.7% in rural areas, 20.7% in urban area, 16.8% in males, and 15.7% in women [7]. In Ethiopia, the second most populous country in Africa, the estimate from meta-analysis showed that the overall prevalence of hypertension was 19.6% and 23.7% in urban area [8]. However, community-based studies revealed that the prevalence of hypertension ranged from 16.9% to 31.5%, and factors such as sociodemographic, economic, biological, and behavioral characteristics were found to be significantly associated with hypertension [9–19]. These evidences showed that the prevalence of hypertension and determinants vary from study to study.

Primary prevention is key to the control of the epidemic of noncommunicable diseases including hypertension, and the identification of major risk factors is important to determine public health priorities [20]. Therefore, knowledge about risk factors should be applied in order to understand the relative effects of sociodemographic, lifestyle (individual behavior), and physiological risk factors comprehensively to integrate intervention efforts across these factors functioning at different level of causation [19].

Previous researches on hypertension treated covariates at different levels as if they all perform at equal level in the causation pathway [9–13] without considering the multidimensional nature of risk factors that are grouped along a causal scale from distant to immediate risk factors. Cognizant of this gap and limited evidence in the study setting, the current study assessed the prevalence and the relative effects of socioeconomic, lifestyle, and physiological (body level) risk factors of elevated blood pressure.

## 2. Methods

Community-based cross-sectional study was conducted from June 01-21, 2017, in Dire Dawa City Administration. Dire Dawa is located at a distance of 515 Kilometers from Addis Ababa, the capital city of Ethiopia. The Dire Dawa administrative council consists of Dire Dawa city and the surrounding rural areas. The council has 4 subcities, 9 urban kebeles (the smallest administrative unit), and 28 rural peasants' associations. According report of Central Statistical Authority (CSA) in 2013, the total population of the administration was 405 444, of which 277 000 were urban population [21].

### 2.1. Population and Sample Size

This study was conducted among 903 adults aged 25-64 years who lived in Dire Dawa for more than six months before the survey. Multistage sampling technique was used; the primary sampling units, five kebeles were randomly selected from the total of nine kebeles; the secondary sampling units, households were selected after the sampling frames were obtained from kebele administrations. Sample size was proportionally distributed to each of the selected kebele based on the number of households in the kebele. Finally, systematic random sampling technique was employed to select households to be visited for data collection. From the selected households, eligible adults aged between 25 and 64 were identified, and if there were more than one in a household, then one was randomly selected or Kish method was used.

### 2.2. Data Collection and Measurement

The data were collected using the WHO STEPS instrument. This survey tool contains three components of risk factor measurement; the first is core and expanded sociodemographic and behavioral characteristics, the second involves core and expanded physical measurement, and the third consisted of biochemical measurements [19]. The tool was translated to Amharic and Afaan Oromo, widely spoken languages in Dire Dawa city Administration. Data were collected by healthcare professionals holding at least BSC degree in nursing after intensive training was given on the objectives of the study, STEPS survey procedures, and tools. Data were collected on weekends and in the afternoon on work days during which time eligible adults were expected to be at home and to balance gender composition at the time of home visit for interview. Before actual data collection started, pre-test was conducted in a kebele that was not included in the study to check for the validity of the instruments and then necessary adjustments were made.

Anthropometric measurement was carried out using standard procedures and the calibrated instruments. Weight was measured using standard digital scale, and stadiometer was used to measure height and the results were recorded to the nearest 0.5 cm. For blood pressure (BP) digital BP apparatus was used. BP was measured three times, 3 to 5 minutes apart from the left arm while the subject was in sitting position and the arm rested on a flat surface. The average of the last two measures was used to determine elevated BP. For biochemical testing, digital glucometer meters were used to measure capillary blood sugar after subject were asked the time lapsed from the last meal.

### 2.3. Operational Definition

WHO and International Diabetic Association (IDA) define diabetes as fasting blood suger≥26 mg/dl or random blood sugar≥200 mg/dl; hypertension: persistently elevated blood pressure, SBP≥140 mmHg or DBP ≥90 mmHg, or reported uses of antihypertensive medication [13, 15, 19, 22]. Ever smoking was defined as smoking cigarette at least one in lifetime. Current alcohol used defined alcohol consumption during the last 30 days preceding. Body mass index was used to classify underweight: BMI<18.5 kg/m^2^, normal BMI: 18.5-24.99 kg/m^2^, and overweight: BMI: ≥25 kg/m^2^; abdominal obesity: waist circumference: male>94 cm and female>80 cm; physical activity was measured as total global physical activity: inactive: <600 MET-minute and active: ≥600 MET-minute.

### 2.4. Data Processing and Analysis

The data were cleared and entered in to EpiData version 3.0 and exported to STATA Version 13.0 statistical software. Separate four regression models were used to show the relative effects of factors associated with elevated blood pressure at different level of risk, from the most distal factors to the nearest causal risk factors [19, 23]. The first model included sociodemographic characteristics. The second model included lifestyle characteristics such as ever tobacco smoking, alcohol consumption, consumption of fruits and vegetables, and exercises. We used ever tobacco smoking as proxy indicator for cigarette smoking because significant among of data value on current smoking status was missing due to non-response. The third model included biophysiological characteristics such as abdominal obesity, BMI, and diabetes. In the final model, all variables were included. Odds Ratio with a 95% confidence interval was presented and a fitness-test was checked for each of the models. Statistical tests were declared significantly at* P*-value<0.05.

### 2.5. Ethical Considerations

Ethical clearance was obtained from the Institutional Health Research Ethics Review Committee, College of Health and Medical Sciences, Haramaya University. Additionally, a permission letter was obtained from Dire Dawa City Administration. Before each interview, informed verbal consent was obtained from respondents. Confidentiality of information was maintained by keeping the anonymity of the individual participants at all levels. Participants with unapparent disease conditions like hypertension and diabetes were referred to nearby health facilities for thorough investigation and prompt management.

## 3. Results

A of total 872 valid observations were included in the analysis. The mean age of participants was 40.334±12.98 years. Five hundred sixty-one (64.34%) of the participants were younger than 45 years old. Two thirds (67.20%) of study participants were female. More than half (52.98%) of adults either attended primary education or did not attend formal education. Two thirds of them (66.74%) were married while 45 (5.16%) were widowed. Four hundred thirteen (47.36%) of the participants were Ethnic Amhara. Concerning occupation, 394 (45.18%) adults were unemployed whereas 234 (26.83%) were employed as office workers (Table 1).

### 3.1. Distribution of Adults' Behavioral Characteristics

From total of participants responding, 74 (8.49%) were lifetime smokers. Two hundred four (23.39%) consumed alcohol over the last 30 days preceding the time of data collection. Three hundred twenty-five (37.27%) participants ate fruits two or fewer days a week. Two hundred seventy-eight (31.88%) participants (38.3%) ate vegetables for two or fewer days during regular week days. Three hundred ninety (44.72%) adults were not involved in adequate physical activity or physical inactivity (Table 2).

### 3.2. Prevalence of Hypertension

**Figure 1:**
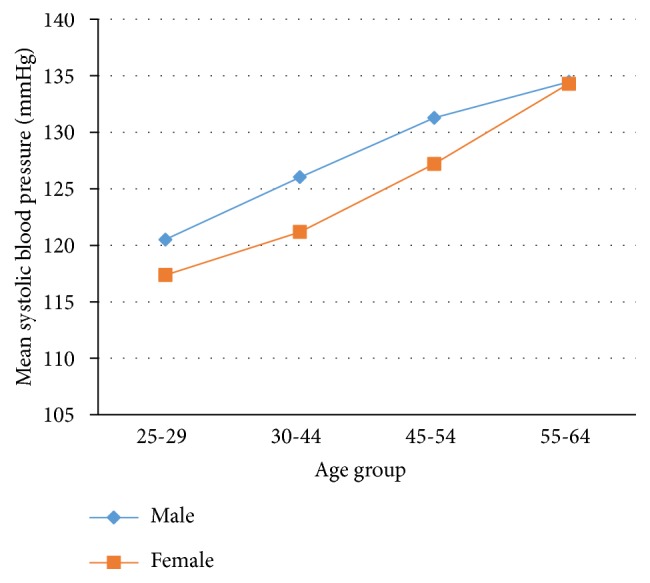
Distribution of mean SBP by age and sex.

**Figure 2:**
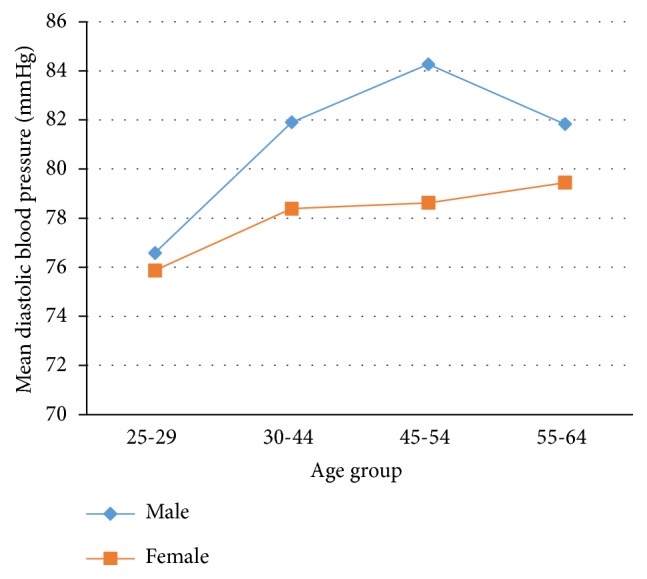
Mean of DBP by age and sex.

The majority, 544 (62.2%) of participants, had checked their blood pressure in the past. The average SBP and DBP was 124.98±17.18 mmHg and 78.92±10.13 mmHg, respectively. The mean SBP was 126.84±16.11 mmHg in men and 124.07±17.61 mmHg in women. However, there was no statistically significant difference in systolic blood pressure between male and female participants (t=1.13 and* P*<0.2575) (Figure 1). The mean DBP was 80.80±11.24 mmHg in men and 78.0±9.42 mmHg in women with statistically significant difference (t=3.85 and P<0.0001) (Figure 2). Both SBP and DBP were significantly increased with age (SBP, F=21.10,* P*<0.001; DBP, F=8.7,* P*<0.001) (Figures 1 and 2). The prevalence of hypertension was 24.43% (95% CI: 21.57, 27.28). More than half (51.64%) adults with hypertension were not aware of their elevated blood pressure at time of the interview.

### 3.3. Distribution of Hypertension by Sociodemographic Features

Slightly higher prevalence of hyertension was observed in male, 73 (25.53%) and it appeared to increases with age in both sex. The prevalence was lowest, (7.33%) among adults 25-29 years-old and the highest, (40.53%) was observed among those 55-64 years-old. The analysis showed that prevalence of hypertension increased with age in both males and females. However, analysis showed that there was not statistically significant difference (*χ*^2^=0.7717,* P*<0.380) between male and female in prevalence of hypertension. The highest prevalence (25.18%) was also observed among ethnic Amhara, divorced (26.39%), no formal education (33.61%), and unemployed (25.90%) adults.

### 3.4. Distribution of Hypertension by Lifestyle and Biophysiological Characteristics

The prevalence of hypertension was higher among lifetime smokers 74 (32.40%), current alcohol consumers 61 (29.90%), physically inactive 104 (26.67%), those having abdominal obesity 127 (31.44%), and diabetic 98 (33.67%), and the prevalence was the highest in adults with BMI≥25 kg/m^2^ 107(31.94%).

### 3.5. Factors Associated with Hypertension

Four logistic regression models were used to illustrate the relative effect of factors associated with hypertension considering risk factors as functioning at different levels. In model-I, hypertension was significantly associated with age group 30-44 (aOR 4.62, 95% CI: 2.52, 8.47), 45-54 (aOR 8.21, 95% CI: 4.12, 16.34), and 55-64 (aOR 12.22, 95% CI: 6.27, 23.83) and being unemployed (aOR 1.65, 95% CI: 1.03, 2.66).

In model-II, lifetime cigarette smoking, current alcohol consumption, total fruit consumption, total vegetable consumption, and physical activity were not associated with hypertension. In the third model, hypertension was significantly associated with abdominal obesity (aOR 1.68, 95% CI: 1.20, 2.36) and BMI≥25 kg/m^2^ (aOR 1.53, 95% CI: 1.08, 2.17).

In the final model, hypertension was significantly associated with age group 30-44 (aOR 3.91, 95% CI: 2.12, 7.24), 45-54 (aOR 6.67, 95% CI: 3.29, 13.49), and 55-64 (aOR 10.54, 95% CI: 5.54, 20.80), being unemployed (aOR 1.68, 95%CI: 1.03, 2.77), ever smoking (aOR 1.89, 95% CI: 1.04, 2.23), abdominal obesity (aOR 1.72, 95% CI: 1.13, 2.64), and BMI≥25 kg/m^2^ (aOR 1.48, 95%CI: 1.01, 2.15) (Table 3).

Overall the analyses revealed that sociodemographic, behavioral, and biophysiological characteristics were significantly associated with hypertension. After controlling for behavioral and biophysiological characteristics, age and unemployment remained strongly associated with hypertension. When adjusted for sociodemographic and biophysiological characteristics, ever smoking, behavioral characteristics turned out to be strongly associated with hypertension.

## 4. Discussion

This study revealed moderately high prevalence and factors associated with hypertension among adults in study setting. The prevalence of hypertension in this study was 24.43%. Alarmingly, the majority of adults (51.64%) were not aware of their elevated blood pressure at time of the interview. This indicates high burden of unapparent morbidity related to hypertension which increases the risk of subsequent complication.

The prevalence of hypertension reported in this study was slightly higher than the results of community-based study conducted in Durame, 22.4% [16], Bedele town, 16.9% [14], and national STEP survey results, 16% [24]. However, the finding of this study was lower than the prevalence reported in study conducted in Bahir Dar City, 25.1% [10], Gondar, 27.4% [15], another study in Gondar [13], Jigjiga 28.3% [12], Hosanna town 30% [11], Dabat, 31.9% [25], and Addis Ababa among civil servants, 27.3% [22]. The observed difference could be due to difference in study population. For instance, unlike our study which was conducted among adults 25-64 years-old, study in Gondar [15, 22] included adults ≥18 years whereas the other study which was conducted in Gondar included adults ≥35 years old. The inclusion of 65 and older might have increased the prevalence of hypertension. The other possible explanation for observed discrepancy could be difference in the definition of hypertension. For example, in national STEP survey [24] definition of hypertension is based only on elevated blood pressure in potentially missing prevalent cases of hypertension on chemotherapy with controlled blood pressure. Additionally, despite attempt to balance gender composition, two thirds (67.20%) of study participants were females in this study which might affect the actual magnitude of hypertension. In line with this argument, literatures [11, 12] showed that prevalence higher among males.

Age was independently associated with hypertension and the odds increased with an increase in age. This can be explained by the fact that with increased age the walls of the larger arteries become stiffened mainly due to arteriosclerotic structural changes, calcification and increased peripheral vascular resistance of smaller arteries [26]. This indicates that hypertension related morbidity increases with increased age along with subsequent complications. The results of this study are consistent with the study conducted in different parts of Ethiopia [9, 10, 13, 15, 22, 25, 27].

Consistent with previous research report [28], unemployed adults had an increased risk of hypertension in the study setting. It was suggested that unemployment may be associated with low socioeconomic status, which in turn reported to have associated with a significant increase in blood pressure [29, 30]. The increased risk of hypertension in unemployed adults could be also the result of lower access to health care services and greater difficulties in adopting health habits [31, 32]. Evidence has also revealed that unemployment is significantly associated with cardiometabolic risk factors such as hypertension and health related quality of life [33].

Cigarette smoking causes activation of the sympathetic nervous system and oxidative stress associated with increase markers of inflammation leading to endothelial dysfunction, vascular injury, plague progression, and increased arterial stiffness leading to development of hypertension [34–38]. Many researchers have reported that cigarette smoking is positively associated with hypertension [10, 18, 22, 39–41] which is similar to the results of our study that revealed lifetime cigarette smoking was significantly associated with hypertension.

Even though pathophysiology is not clear, abdominal obesity is an important public health problem associated with hypertension, disability, and higher mortality among older adults [42–44]. The growing evidence showed abdominal obesity independent of body mass index or generalized obesity [43, 45]. Consistent with our study, the study in Ecuador among older adults [46] revealed that hypertension was significantly associated with abdominal obesity. However, a study conducted among adults in Jigjiga City [12] reported that abdominal obesity was not associated with hypertension. The difference could be attributed to gender balance as it was nearly equal in study conducted in Jigjiga whereas in our study two thirds of the participants were females even though there was no significant association with hypertension. The other explanation for the observed discrepancy could be attributed to difference in sample size. In fact, the effect of abdominal obesity is not well documented in Ethiopian context.

Even though some argue that BMI along is not good predictor of cardiovascular disease indicating that adverse health consequences are associated with increased adiposity rather than an increase in body weight [47, 48], evidence showed that higher BMI accounts for 75% of the risk of primary hypertension which is intermediated by increased renal tubular sodium reabsorption that impairs natriuresis [49]. Several community-based studies conducted in different parts of Ethiopia [9, 10, 12, 13, 15, 16, 22, 27, 50, 51] revealed BMI≥25 kg/m^2^ associated with hypertension. Similar observation has been made in our study revealing that having higher BMI was associated with hypertension.

## 5. Limitation

While sharing the methodological limitations of cross-sectional studies, additionally, the effects' family history of hypertension, other eating behaviors other than fruits and vegetables, khat consumption, and lipid profile were not assessed which could have been associated with elevated blood pressure. Lastly, males were underrepresented unintentionally which may have resulted in a biased estimate.

## 6. Conclusion and Recommendation

Moderately high prevalence of hypertension was noted among adults in study setting demonstrating a major public health problem. The majority of adults were unaware of their elevated blood pressure highlighting the burden of the hidden morbidity which could accelerate risk of subsequent complications such as end organ damage. Therefore, community intervention directed towards changing or modifying behavioral risk factors should be developed to address individual level risk factors. Additionally, routine assessment of sociodemographic, behavioral, and biophysiological risk factors, screening, and diagnosis of NCDs should be institutionalized to address the occult burden. Furthermore, future prospective research should accurately identify the sociodemographic, behavioral, and biophysiological level risk factors of elevated blood pressure in the Ethiopian context.

## Figures and Tables

**Table 1:** Sociodemographic characteristics of adults in Dire Dawa city, 2017.

Characteristics	Frequency	Percent
Sex		
Male	286	32.80
Female	586	67.20

Age		
25-29	232	26.61
30-44	329	37.73
45-54	121	13.88
55-64	190	21.79

Education		
College and above	101	13.99
Secondary	309	35.44
Primary	340	38.99
No education	122	13.99

Marital status		
Married	582	66.74
Single	173	19.84
Divorced	72	8.26
Widowed	45	5.16

Ethnicity		
Oromo	301	34.52
Amhara	413	47.36
Other^†^	158	18.12

Occupation		
Office work	234	26.83
Merchant	135	15.48
Unemployed	394	45.18
Other^††^	109	12.50

Other^†^: including Somali (n=28), Harari (n=10), Gurage (n=55), Tigre (n=39), Wolayta (n=8), Silte (=15), and Afar (n=3).

Other^††^: including housewife (n=36), pensioner (n=30), janitor (n=13), and daily laborer (n=30).

**Table 2:** Distribution of adults' behavioral characteristics in Dire Dawa, 2017.

Tobacco status	Frequency	Percentage
Ever smoking cigarette		
Yes	74	8.49
No	798	91.51

Current alcohol consumption		
Yes	204	23.39
No	668	76.61

Fruits consumption per week		
Two or fewer	325	37.27
Three to four	190	21.79
Five or more	357	40.94

Vegetables consumption per week		
Two or fewer	278	31.88
Three to four	235	26.95
Five or more	359	41.17

Total physical activities		
Active	482	55.28
Inactive	390	44.72

**Table 3:** Factors associated with hypertension in Dire Dawa City Administration, 2017.

Characteristics	Model-I	Model-II	Model-III	Final model
Sex				
Male	1.18 (0.82, 1.74)	-	-	1.39(0.88, 2.20)
female	1.00	-	-	1.00

Age				
25-29	1.00			1.00
30-44	4.62(2.52, 8.47)^**∗∗**^	-	-	3.91(2.12, 7.24)*∗∗*
45-54	8.21(4.12, 16.34)^**∗∗**^	-	-	6.67(3.29, 13.49)*∗∗*
55-64	12.22(6.27, 23.83)^**∗∗**^	-	-	10.64(5.35, 20.80)*∗∗*

Education				
Degree+	1.54(0.73, 3.29)	-	-	1.52(0.70, 3.31)
Secondary	1.31(0.74, 2.31)	-	-	1.21(0.67, 2.17)
Primary	0.79(0.47, 1.31)	-	-	0.76(0.45, 1.29)
No education	1.00	-	-	1.00

Marital status				
Married	1.00	-	-	1.00
Never married	1.07(0.65, 1.76)	-	-	1.14(0.68, 1.90)
Divorced	0.90(0.50, 1.62)	-	-	0.92(0.50, 1.70)
Widowed	0.49(0.23, 1.05)	-	-	0.48(0.22, 1.05)

Ethnicity				
Oromo	1.00	-	-	1.00
Amhara	0.98(0.66, 1.45)	-	-	0.97(0.64, 1.46)
Other^†^	1.19(0.73, 1.97)	-	-	1.14(0.67, 1.93)

Occupation				
Office worker	1.00	-	-	1.00
Unemployed	1.65(1.03, 2.66)*∗*	-	-	1.68(1.03, 2.77)*∗*
Merchant	1.52(0.88, 2.61)	-	-	1.48(0.85, 2.58)
Other^††^	1.09(0.59, 1.99)	-	-	0.99(0.53, 1.86)

Smoking tobacco				
Yes	-	1.58(0.94, 2.67)	-	1.89(1.04, 3.45)*∗*
No	-	1.00	-	1.00

Current alcohol use				
Yes	-	1.37(0.93, 2.02)	-	1.41(0.89, 2.23)
No	-	1.00	-	1.00

Fruit consumption/wk				
2 or fewer days	-	1.04(.064, 1.71)	-	1.16(0.66, 2.04)
3-4 days	-	0.95(0.57, 1.60)	-	1.02(0.56, 1.85)
5 or more days	-	1.00	-	1.00

Vegetables consumption				
2 or fewer days	-	1.07(0.65, 1.77)	-	1.03(0.59, 1.79)
3-4 days	-	1.02(0.63, 1.66)	-	1.04(0.63, 1.74)
5 or more days	-	1.00	-	1.00

Physical activity				
Active	-	1.00	-	1.00
Inactive	-	1.29(0.94, 1.78)	-	1.11(0.78, 1.59)

Abdominal obesity				
Yes	-	-	1.68(1.20, 2.36)*∗*	1.72(1.13, 2.64)*∗*
No	-	-	1.00	1.00

BMI (kg/m^2^)				
18.5-24.9	-	-	1.00	1.00
<18.5	-	-	0.69(0.33, 1.47)	0.67(0.30, 1.50)
≥25	-	-	1.53(1.08, 2.17)*∗*	1.48(1.01, 2.15)*∗*

Diabetes				
Yes	-	-	1.51(0.95, 2.40)	1.22(0.73, 2.03)
No	-	-	1.00	1.00

1.00: reference category; *∗*: *P*<0.05, *∗∗*: *P*<0.000; other^†^: including Somali (n=28), Harari (n=10), Gurage (n=55), Tigre (n=39), Wolayta (n=8), Silte (=15), and Afar(n=3); other^††^: including housewife (n=36), pensioner (n=30), janitor (n=13), and daily laborer (n=30).

## Data Availability

Contact correspondent authors through e-mail if there is need for database.
